# Quality of inter-hospital transportation in 431 transport survivor patients suffering from acute respiratory distress syndrome referred to specialist centers

**DOI:** 10.1186/s13613-018-0357-y

**Published:** 2018-01-15

**Authors:** Sebastian Blecha, Frank Dodoo-Schittko, Susanne Brandstetter, Magdalena Brandl, Michael Dittmar, Bernhard M. Graf, Christian Karagiannidis, Christian Apfelbacher, Thomas Bein, For the DACAPO Study Group, Johannes Bickenbach, Thorben Beeker, Tobias Schürholz, Jessica Pezechk, Jens Schloer, Ulrich Jaschinski, Ilse Kreuzer, Oliver Kuckein, Steffen Weber-Carstens, Anton Goldmann, Stefan Angermair, Krista Stoycheva, Jörg Brederlau, Nadja Rieckehr, Gabriele Schreiber, Henriette Haennicke, Friedhelm Bach, Immo Gummelt, Silke Haas, Catharina Middeke, Ina Vedder, Marion Klaproth, Michael Adamzik, Jan Karlik, Stefan Martini, Luisa Robitzky, Christian Putensen, Thomas Muders, Ute Lohmer, Rolf Dembinski, Petra Schäffner, Petra Wulff-Werner, Elke Landsiedel-Mechenbier, Daniela Nickoleit-Bitzenberger, Ann-Kathrin Silber, Maximilian Ragaller, Marcello Gama de Abreu, Alin Ulbricht, Linda Reisbach, Kai Zacharowski, Patrick Meybohm, Karin Pense, Gerhard Schwarzmann, Johannes Reske, Alexander Hötzel, Johannes Kalbhenn, Christoph Metz, Stefan Haschka, Stefan Rauch, Michael Quintel, Lars-Olav Harnisch, Sophie Baumann, Andrea Kernchen, Sigrun Friesecke, Sebastian Maletzki, Stefan Kluge, Olaf Boenisch, Daniel Frings, Birgit Füllekrug, Nils Jahn, Knut Kampe, Grit Ringeis, Brigitte Singer, Robin Wüstenberg, Jörg Ahrens, Heiner Ruschulte, Andre Gerdes, Matthias Groß, Olaf Wiesner, Aleksandra Bayat-Graw, Thorsten Brenner, Felix Schmitt, Anna Lipinski, Dietrich Henzler, Klaas Eickmeyer, Juliane Krebs, Iris Rodenberg, Heinrich Groesdonk, Kathrin Meiers, Karen Salm, Thomas Volk, Stefan Fischer, Basam Redwan, Martin Schmölz, Kathrin Schumann-Stoiber, Simone Eberl, Gunther Lenz, Thomas von Wernitz-Keibel, Monika Zackel, Frank Bloos, Petra Bloos, Anke Braune, Anja Haucke, Almut Noack, Steffi Kolanos, Heike Kuhnsch, Karina Knuhr-Kohlberg, Markus Gehling, Mathias Haller, Anne Sturm, Jannik Rossenbach, Dirk Schädler, Stefanie D’Aria, Christian Karagiannidis, Stephan Straßmann, Wolfram Windisch, Thorsten Annecke, Holger Herff, Michael Schütz, Sven Bercker, Hannah Reising, Mandy Dathe, Christian Schlegel, Katrin Lichy, Wolfgang Zink, Jana Kötteritzsch, Marc Bodenstein, Susanne Mauff, Peter Straub, Christof Strang, Florian Prätsch, Thomas Hachenberg, Thomas Kirschning, Thomas Friedrich, Dennis Mangold, Caroline Rolfes, Tilo Koch, Hendrik Haake, Katrin Offermanns, Patrick Friederich, Florian Bingold, Michael Irlbeck, Bernhard Zwissler, Ines Kaufmann, Ralph Bogdanski, Barbara Kapfer, Markus Heim, Günther Edenharter, Björn Ellger, Daniela Bause, Götz Gerresheim, Dorothea Muschner, Michael Christ, Arnim Geise, Martin Beiderlinden, Thorsten Heuter, Alexander Wipfel, Werner Kargl, Marion Harth, Christian Englmeier, Thomas Bein, Sebastian Blecha, Kathrin Thomann-Hackner, Marius Zeder, Markus Stephan, Martin Glaser, Helene Häberle, Hendrik Bracht, Christian Heer, Theresa Mast, Markus Kredel, Ralf Müllenbach

**Affiliations:** 1 Department of Anaesthesiology, University Medical Centre Regensburg, Franz-Josef-Strauss-Allee 11, 93053 Regensburg, Germany; 2 Medical Sociology, Institute of Epidemiology and Preventive Medicine, University of Regensburg, Dr.-Gessler-Str. 17, 93051 Regensburg, Germany; 3 Department of Pneumology and Critical Care Medicine, Cologne-Merheim Hospital, ARDS and ECMO Centre, Kliniken der Stadt Köln gGmbH, Witten/Herdecke University Hospital, Ostmerheimer Strasse 200, 51109 Cologne, Germany

**Keywords:** Inter-hospital transfer, ARDS, Quality of care

## Abstract

**Background:**

The acute respiratory distress syndrome (ARDS) is a life-threatening condition. In special situations, these critically ill patients must be transferred to specialized centers for escalating treatment. The aim of this study was to evaluate the quality of inter-hospital transport (IHT) of ARDS patients.

**Methods:**

We evaluated medical and organizational aspects of structural and procedural quality relating to IHT of patients with ARDS in a prospective nationwide ARDS study. The qualification of emergency staff, the organizational aspects and the occurrence of critical events during transport were analyzed.

**Results:**

Out of 1234 ARDS patients, 431 (34.9%) were transported, and 52 of these (12.1%) treated with extracorporeal membrane oxygenation. 63.1% of transferred patients were male, median age was 54 years, and 26.8% of patients were obese. All patients were mechanically ventilated during IHT. Pressure-controlled ventilation was the preferred mode (92.1%). Median duration to organize the IHT was 165 min. Median distance for IHT was 58 km, and median duration of IHT 60 min. Forty-two patient-related and 8 technology-related critical events (11.6%, 50 of 431 patients) were observed. When a critical event occurred, the PaO_2_/FiO_2_ ratio before transport was significant lower (68 vs. 80 mmHg, *p* = 0.017). 69.8% of physicians and 86.7% of paramedics confirmed all transfer qualifications according to requirements of the German faculty guidelines (DIVI).

**Conclusions:**

The transport of critically ill patients is associated with potential risks. In our study the rate of patient- and technology-related critical events was relatively low. A severe ARDS with a PaO_2_/FiO_2_ ratio < 70 mmHg seems to be a risk factor for the appearance of critical events during IHT. The majority of transport staff was well qualified. Time span for organization of IHT was relatively short. ECMO is an option to transport patients with a severe ARDS safely to specialized centers.

*Trial registration* NCT02637011 (ClinicalTrials.gov, Registered 15 December 2015, retrospectively registered)

## Background

The acute respiratory distress syndrome (ARDS) is a life-threatening condition characterized by either direct or indirect damage to the lung parenchyma often causing critical hypoxemia and/or hypercapnia [1]. The mortality for ARDS remains high between 30 and 60% despite all the progress made in intensive care during the last decades [2, 3]. The management of this severe disease at specialized intensive care units (ICU) is a resource-consuming process that requires a specially qualified team, a high-technology standard and sophisticated treatment strategies to provide life-sustaining therapy [4]. Healthcare research in the field of critical care medicine is relatively new, and data on the influence of organizational structures or processes of care on mortality or health-related quality of life (HRQoL) in ICU survivors are of growing interest [5, 6]. Not all of these critically ill patients with an ARDS are treated primarily in a specialized center and, thus, eventually experience inter-hospital transfer (IHT).

In Germany, the frequency of IHT has grown considerably in recent years and will steadily increase as a result of the expansion of specialized centers. For example, in the southwestern part of Germany the number of IHTs rose by 9.4% from 2014 to 2015 [7]. As a consequence, the health expenditures for patient transports were increased each year and for Germany overall costs rose to 5.94 million Euros in 2013 [8].

The IHT is a secondary transport from one institution of initial or standard care for further therapy to an institution of advanced and/or maximum care, while maintaining the intensive medical therapy already in progress [9, 10]. In this context, the IHT is synonymous with the term intensive care transport. The decision to transport an ARDS patient is based on an assessment of the potential benefits of transport weighed against the potential risks. The transport must be as safe as possible and should not pose additional risks.

In Germany, IHT is conducted regularly by the authorized organizations of the Emergency Medical Services (EMS) coordinated by the local Emergency Medical Dispatch Center (EMDC). Standard EMS vehicles do not provide special intensive care equipment. ARDS patients require continuous monitoring and expanded intensive medical equipment (e.g., for lung-protective mechanical ventilation) and accordingly staff trained in intensive care medicine. These critically ill patients should be transferred with special vehicles such as intensive care ambulances (ICA) or intensive care helicopters (ICH).

In this study, we assessed the quality of IHT in transport survivor patients suffering from ARDS referred to specialized centers in a Germany-wide multicenter study. In addition to the means of transport used and the transport duration, the qualification of transportation personnel, technical equipment, ventilation parameters and the incidence of critical events were recorded.

## Methods

### Study design and sample

We observed the quality of IHT in patients with an ARDS in the context of a large Germany-wide prospective cohort study (DACOPO study [11], ClinicalTrials.gov Identifier: NCT02637011). Briefly, the DACAPO study investigates the influence of quality of care and individual patient characteristics on HRQoL and return to work in survivors of ARDS. The study has been approved by the Ethics Committee of the University of Regensburg (original approval: December 2013, approval of an amendment: June 2014; file number 13-101-0262) and (if necessary) by Ethics Committees of the participating study centers. The inclusion criteria for enrollment of patients were the presence of an ARDS according to the Berlin definition [12] and being 18 years of age or older. Written informed consent was obtained from caregivers/legal guardians and additionally from those patients who survived ICU. ARDS patients were included in the study after admittance to one of the participating hospitals, and data on transport were collected retrospectively. Mortality during transportation could thus not be analyzed. Eligible patients were enrolled in 34 receiving hospitals all over Germany in the period from September 2014 to April 2016 (*N* = 1234). The health status of all transferred patients who had been treated before in a referring hospital was retrospectively assessed for the period of their stay in this hospital and for the period during inter-hospital transport. In the following, the examined aspects of the structural-, process- and outcome-related quality with regard to IHT are explained.

### Measures

#### Sociodemographic and clinical parameters

The following sociodemographic and clinical parameters were assessed: age, body mass index (BMI), distribution of severity of ARDS, cause of ARDS, applied oxygen fraction (FiO_2_), positive end-expiratory pressure (PEEP), concentration of applied continuous infusion of norepinephrine (noradrenalin), sequential organ failure assessment (SOFA), use and duration for implementation of extracorporeal membrane oxygenation (ECMO) and type of used respirator. The clinical parameters of patients before transport were stated in the morning of the transportation day.

#### Structural quality of IHT

The structural quality of IHT can be determined on the basis of technical and human resources. In Germany, technical equipment of rescue vehicles is standardized by Germany’s national standards body (DIN norms) and European Committee for Standardization (CEN norms), respectively. The qualification of the EMS staff for IHT is recommended by the provisions of the German Interdisciplinary Association of Critical and Emergency Care Medicine (DIVI: Deutsche Interdisziplinäre Vereinigung für Intensiv- und Notfallmedizin) and German Confederation of Associations of Emergency Physicians (BAND e.V.: Bundesvereinigung der Arbeitsgemeinschaften der Notärzte Deutschlands e.V.) [13].

##### Technical equipment of EMS vehicles

For transporting patients, specific standards are required. Three types of ambulances for the ground-based EMS vehicles with a minimum of technology standard are available in accordance with CEN 1789:2014-12:

- Type A: Patient Transport Ambulance
- Type B: Emergency Ambulance
- Type C: Mobile Intensive Care Unit

In addition, the German EMS is equipped with a limited number of specialized ICA, which is designated for IHT of critically ill patients. The specifications for ICA are described in DIN 75076:2012-05.

For the airborne transportation of patients, emergency helicopters or ICH are available. The technical standards of medical helicopters are normed by CEN 13718-1:2014 (Medical vehicles and their equipment—Air ambulances—Part 1: Requirements for medical devices used in air ambulances) and CEN 13718-2:2015 (Medical vehicles and their equipment—Air ambulances—Part 2: Operational and technical requirements for air ambulances). In Germany, some helicopters are dedicated to dual use, i.e., these air ambulances can be used in the regular EMS as well as in intensive care transport [14].

Based on these standards, we assessed the following items (yes/no): type of EMS vehicles and problems with technical equipment.

##### Qualification of transportation staff

For the escorting physician, we assessed the following items (yes/no), based on DIVI recommendations [13, 14]:

1. Three years of clinical training in a field with intensive care tasks,
2. Six months verifiable full-time on an ICU,
3. Qualification as a pre-hospital emergency physician in the EMS according to local regulations,
4. Attending a 20-h course “intensive care transportation”

Due to the specific requirements of intensive care transports, the responsible paramedic also must have an additional qualification [13]. We assessed the following items (yes/no):

1. Professional qualification: paramedic
2. At least 3 years in an emergency service in a full-time or a time comparable to professional experience
3. Attending a 20-h course “intensive transport for emergency services personnel”

#### Process quality of IHT

The planned and used EMS vehicle, the IHT distance, the duration of the planning of IHT and the actually required duration of IHT were recorded using a specific case report form during patient admission in the receiving hospital and were extracted from the DIVI transport protocols [15, 16]. The communication between the referring and receiving hospital and between referring and transport physician was also registered. In the planning of the study, we decided not to register respirator settings (tidal volume, breathing frequency) or lung-protective ventilation, because these parameters were too variable and not documented in a standardized manner.

#### Outcome-related quality of IHT

Changes in oxygenation (PaO_2_/FiO_2_) and critical events (cardiopulmonary resuscitation, accidental extubation, hypoxia, and hypotension) were considered indicators of the outcome-associated qualities during IHT.

#### Data collection

The clinical data from the referring clinics were recorded by means of a fax request. Furthermore, communication between physicians of the referring and receiving hospitals was registered. For each transferred patient, 220 up to 295 items were collected from study participants in the receiving hospitals. The collected data were transferred to the online documentation system (OpenClinica^®^) as comprehensive as possible. In addition, the receiving physicians recorded whether an ARDS was overlooked in patients who were transferred.

#### Statistical analysis

Extensive plausibility tests were performed to ensure data quality. Due to the explorative character of this study, imputation methods are not appropriate. Thus, the statistical analyses are based on complete data for each parameter. Descriptive statistics were performed by calculating median (Md) and inter-quartile range (IQR) for continuous variables. Counts and percentages were calculated for categorical variables. For inference statistical analyses regarding central tendency, Wilcoxon signed-rank test (dependent samples) and the Mann–Whitney *U* test (independent samples) were carried out. In the case of categorial data, Fisher’s exact test was used. All tests are two-tailed. The significance level was set at *p* < 0.05. Analyses were computed using IBM SPSS Statistics (version 24).

## Results

A total of 431 patients with an ARDS were transferred from 315 different referring hospitals into 34 different receiving hospitals. Twenty-nine of these 34 hospitals (85%) are members of the German ARDS network. The median treatment time was 4 days (IQR 2–8) in the primary hospital before transportation. In median, six ARDS patients from five different hospitals were transferred per receiving hospital. In addition, the physicians of the receiving hospitals registered in 49 cases that the ARDS has been overlooked in the referring clinic in terms of a missing diagnosis. These 49 cases could not be included in the analysis.

### Sociodemographic and clinical parameters

The median age of ARDS patients was 54 years (IQR 43.0–64.0). 272 of the patients (63.1%) were male. In 84%, direct pulmonary injury was the cause of ARDS. This ARDS cohort had a median BMI of 27.8 kg/m^2^ (IQR 24.7–33.4), and 26.8% were obese with BMI ≥ 30 kg/m^2^. The patients’ clinical characteristics before and after transportation are shown in Table 1. Particularly, patients with a moderate or severe ARDS were transported. The dose of noradrenalin infusion was stable during the IHT. All patients were mechanically ventilated during transport, predominantly in a pressure-controlled manner (81.3% biphasic positive airway pressure [BIPAP], 10.8% pressure control ventilation [PCV]). The types of respirators used were heterogeneous: the Oxylog 3000 plus (Fa. Dräger^®^) was most frequently mentioned (39%). The ventilation of the ARDS patients during transport was performed with a median positive end-expiratory pressure (PEEP) of 15 cm H_2_O under applied FiO_2_ of 0.9 (median). In total, 52 patients (12.3%) with median age of 52.5 years were transferred while being treated with ECMO. The implantation of ECMO was carried out in the referring hospital (median 60 min before IHT) by specialized ECMO teams.

**Table 1:** Patient characteristics before and after transport (Wilcoxon test)

	Before transport^a^	After transport^b^	*p* value
ARDS severity (*n* = 295) (according to Berlin definition)			
Mild (*n*/%)	4/1.3	13/4.4	
Moderate (*n*/%)	69/23.4	97/32.9	
Severe (*n*/%)	222/75.3	185/62.7	
SOFA score (*n* = 147) median (IQR)	10 (8–12)	11 (8–13)	< 0.001*
PaO_2_/FiO_2_ (mmHg)			
All patients (*n* = 293) median (IQR)	78.8 (62.9–100)	82.9 (64.6–121.9)	0.002*
Without ECMO (*n* = 251) median (IQR)	80.0 (63.3–101.3)	81.5 (63.8–115.1)	0.059
With ECMO (*n* = 42) median (IQR)	72.5 (54.1–92.1)	95.6 (67.9–131.7)	0.002*
Continuous infusion of noradrenalin (µg/kg/min) (*n* = 173) median (IQR)	0.22 (0.10–0.59)	0.24 (0.11–0.59)	0.388

^a^In the morning of the transportation day

^b^Immediately after ICU admission in the receiving hospital

* *p* < 0.05

### Structural quality of IHT

#### Technology-related problems of EMS vehicles

In our study, we observed eight technology-related problems. Equipment problems comprised especially the respirator and syringe pump. In three cases a change in EMS vehicle was necessary (Table 2).

**Table 2:** Frequency of critical events in 431 observed patients

Critical events	Total (*n*)
Patient-related	
Cardiopulmonary resuscitation	3
Accidental extubation	2
Hypoxia (SpO_2_ < 85% over at least 5 min)	30
Hypotension (systolic blood pressure < 80 mmHg over at least 5 min)	7
Technology-related	
Equipment problems (thereof: respirator/syringe pump/others)	5 (3/1/1)
Transportation vehicle change necessary	3
Transportation delay	
Occurred transportation delay^a^ (thereof: weather-/staff-related problems/others)	9 (3/1/5)

^a^Survey of transport physician by receiving physician

#### Qualification of transportation staff

The qualification of the transportation staff according to the requirements of the DIVI is summarized in Table 3. All transport teams included a physician. Nearly three-quarter of the physicians attended the intensive transport course.

**Table 3:** Qualification of transportation staff

Qualification of transportation staff	Results *n* (%)	Unknown/missing *n*
Transport physician		
3 years of clinical training in a field with intensive care tasks with additional 6 months verifiable full-time in an ICU	256 of 279 (91.8)	152
Qualification as an EMS physician (according to local regulations)	226 of 237 (95.4)	194
20-h course “intensive transportation”	131 of 179 (73.2)	252
→ All DIVI qualifications	125 of 179 (69.8)	252
Paramedic		
≥3 years in an emergency service in a full-time or a time comparable to professional experience	188 of 190 (99.0)	241
20-h course “intensive transport for emergency services personnel”	144 of 166 (86.7)	265
→ All DIVI qualifications	144 of 166 (86.7)	265

### Process quality of IHT

#### Transport characteristics

The median time interval for inter-hospital transport of ARDS patients between request of the referring doctor to the local EMDC and the start of the patient transport was 165 min (IQR 90–290 min). The median time span to organize the IHT was 180 min for airborne and 160 min for ground-based transportation. There were no significant differences in patient characteristics between ground-based and airborne transportation. Over two-thirds of the ARDS patients were transported on the ground; in nearly one-third of all patients an airborne transport was operated. The median distance between the referring and receiving hospitals was 58 km (IQR 23–105 km), and the median transport time was 60 min (IQR 40–90 min). The median duration of transport for patients using ECMO was also 60 min. The median distance and duration for ground-based transportation were 37 km in 60 min compared to 99 km in 54 min for airborne transport. Table 4 compares the transport vehicle requested by the referring institution and the ones actually used. No patient was transferred by EMS vehicle Type A. Transportation delays were caused by weather- and staff-related problems.

**Table 4:** Characteristics of rescue transport vehicle used (requested and actually used)

Transport vehicle	By referring hospital requested vehicle *n* (%)	Used vehicle *n* (%)
Emergency helicopter/Intensive care helicopter	120 (35.7)	115 (32.2)
Intensive care ambulance	177 (52.7)	191 (53.5)
Emergency ambulance (type B/C CEN 1789:2014)	39 (11.6)	51 (14.3)
Total	336 (100)	357 (100)
Unknown/missing	95 of 431 (22.0)	74 of 431 (17.2)

#### Communication

In 99% (388 of 392 cases, 39 missing data) of transfers, direct communication took place between the referring and receiving hospitals. In four cases the receiving hospital was not aware of the patient allocation before the beginning of IHT. A structured conversation about the patient’s condition between referring and transport physician took place in 99.1% (345 of 348 cases, 83 missing data).

### Outcome quality of IHT

For all patients who oxygenation data were available, the median PaO_2_/FiO_2_ ratio was significantly better after IHT (*p* = 0.002) (Table 1). This applies also to patients with ECMO (*p* = 0.002). Patients with ECMO tended to have a better PaO_2_/FiO_2_ ratio after transport than patients without ECMO. A change in the severity of ARDS before and after transport was observed in 76 patients. After transportation only 62.7% had an ARDS classified as “severe” as compared to 75.3% before transportation (Table 1). During transport, 42 patient-related and 8 technology-related critical events (11.6%, 50/431 patients) were observed (Table 2). Neither the qualification of the transportation staff nor the used transport vehicle was associated with the occurrence of critical events (*p* = 0.14). However, the median PaO_2_/FiO_2_ ratio before transport was significantly lower in patients who experienced a critical event during the transport (Md = 68 mmHg vs. Md = 80 mmHg, *p* = 0.017). The occurrence of hypoxia during IHT (*n* = 30) was lower in patients under ECMO compared to patients without ECMO [3.8% (2 of 52 cases) vs. 7.4% (28 of 379 cases)].

## Discussion

This is the first study reporting data from a large number of ARDS patients, who had to be transferred predominantly to specialized hospitals in Germany. The IHT data of 431 patients were investigated prospectively.

The main results of our study are: (1) in most cases transport of ARDS patients was safe and without serious organ failure; (2) process quality for IHT organization: the elapsed time between the request and the departure of the transport was relatively short (Md = 165 min), median duration of transport was 60 min, 68% ground-based transport; (3) sufficient quality of outcome: critical events were rare, no deterioration of gas exchange, no change in concentration of infused catecholamine; (4) good structural quality: the majority of the IHT staff possessed specific qualifications.

The severity of hypoxemia (PaO_2_/FiO_2_ ratio ≤ 100 mmHg) is an important predictor for mortality in ARDS patients [12], and hypoxemia is one of the reasons to transfer a patient into a specialized center. In this study the majority of ARDS patients (85%) were transported to ARDS/ECMO centers for further treatment. Patients with a severe ARDS and potentially reversible respiratory failure may have a better chance to survive when transported to an ECMO center [17–19].

Warren et al. [20] claimed in their guidelines that all critical care transports ideally should be performed by specially trained professionals and the development of ECMO retrieval teams may lead to a safer transport of ARDS patients to tertiary care centers [21]. In this study the qualification of the transportation staff according to the requirements of the DIVI and BAND was respectable, but physician’s participation on DIVI intensive transportation courses might be improved.

In a populous country like Germany, ARDS patients were transferred in a median of 60 min over a distance of 58 km. In our study, patients were transported mainly earth-bound. The airborne transportation of ARDS patients seems to be faster over longer transport distance, but the time span for organization was longer. The use of ECMO during the transport showed no relevant influence on the duration of transport. In a US study in the State of New York, 17 patients with ECMO were transported in 60 min over a mean distance of 37 km [22]. An Italian mobile ECMO team in Monza transferred 42 patients over 121 km in a mean mission time of 508 min [21]. A Canadian study showed no influence of transport times on hospital mortality [23].

The ventilation settings during IHT could have an influence on patients’ outcome. ARDS patients receiving higher PEEP levels (mean difference of at least 3 cm H_2_O between groups) had a trend toward improved survival [24]. But high PEEP can conceivably cause ventilator-induced lung injury, pneumothorax or decreased cardiac output. In our study, an oxygen fraction of 0.9 was chosen by the majority presumably to avoid hypoxemia during IHT. PCV for ARDS patients is associated with lower peak airway pressures, a more homogeneous gas distribution, improved patient-ventilator synchrony and earlier liberation from mechanical ventilation compared to volume-controlled ventilation [25, 26]. The use of PCV combined with adequate median PEEP of 15 mbar seems to be an indicator for a good quality of care during IHT.

ECMO treatment for transportation of severely hypoxic patients may allow safe and controlled transport to maximum care institutions. Isgrò et al. [27] showed in a 5-year report of 12 Italian patients with severe ARDS that ECMO effectively enabled high-risk ground transfer, significantly improved the arterial oxygenation and allowed a protective lung ventilation. In a Korean study, 18 patients with ECMO therapy were transferred without ventilation to a specialized center [28]. In our study in 52 patients with severe ARDS ECMO cannulations were performed by specialized teams in the referral hospitals and they were transported to receiving hospitals.

Nevertheless, the transport of critically ill patients remains risky. A large Canadian study [29] as well as Rosenberg et al. [30] showed for critically ill transported patients an increased odds ratio of ICU death relative to patients admitted directly to ICU from the emergency room. In the CESAR trial [31], 81 patients from the ECMO group were transported on mechanical ventilation and 2 of these patients (2.4%) died during transport. In our study we observed 42 patient-related and 8 technology-related critical events (11.8%). This incidence might be relatively low, especially considering the possibility of transfer to specialized ARDS centers. Additionally in another study the rate of adverse events during IHT was reported to be low [32]. In the Netherlands the use of a specialist retrieval team reduced the occurrence of adverse events from 34 to 12.5% [33]. Nevertheless, a severe ARDS with a PaO_2_/FiO_2_ ratio < 70 mmHg is associated with an elevated risk for the appearance of critical events during IHT.

### Strengths and limitations

Our study has some limitations. First, in patients, where an existing ARDS was not diagnosed by referring hospital, no information was available about the quality of transport. The IHT data were collected Germany-wide by different investigators. Unfortunately, we lack information about the type of ECMO (veno-venous, veno-arterial). Furthermore, due to the complex procedures of obtaining written informed consent, we could only include ARDS patients after transportation to the participating hospitals of the DACAPO study.

The strengths of our study are: a pre-specified protocol and case report forms, data collection in several centers across Germany, all elements of quality (structure-, process-, outcome-associated) were assessed.

## Conclusion

The transport of critically ill patients is associated with certain risks. The majority of transport staff in our study was well qualified. The rate of patient- and technology-related critical events was relatively low. The time span between the initiation of the transfer procedure and the realization was good and better than expected. Our data suggest that ECMO is an option to transport patients with a severe ARDS safely to specialized ECMO centers, ideally by trained retrieval teams.

## Abbreviations

ARDS: acute respiratory distress syndrome
BMI: body mass index
ECMO: extracorporeal membrane oxygenation
EMS: Emergency Medical Services
EMDC: Emergency Medical Dispatch Center
FiO_2_: inspiratory fraction of oxygen
HRQoL: health-related quality of life
ICA: intensive care ambulances
ICH: intensive care helicopters
ICU: intensive care unit
IHT: inter-hospital transfer
PEEP: positive end-expiratory pressure
SOFA: sequential organ failure assessment
